# Risk perception and behaviour during the COVID-19 pandemic: Predicting variables of compliance with lockdown measures

**DOI:** 10.1371/journal.pone.0262319

**Published:** 2022-01-05

**Authors:** Sara Lo Presti, Giulia Mattavelli, Nicola Canessa, Claudia Gianelli

**Affiliations:** 1 IUSS Cognitive Neuroscience (ICON) Center, Scuola Universitaria Superiore IUSS, Pavia, Italy; 2 Istituti Clinici Scientifici Maugeri IRCCS, Cognitive Neuroscience Laboratory of Pavia Institute, Pavia, Italy; Fondazione Istituto Neurologico Nazionale C Mondino Istituto di Ricovero e Cura a Carattere Scientifico, ITALY

## Abstract

The COVID-19 pandemic and the measures to counteract it have highlighted the role of individual differences in evaluating and reacting to emergencies, and the challenges inherent in promoting precautionary behaviours. We aimed to explore the psychological and cognitive factors modulating behaviour and intentions during the national lockdown in Italy. We administered an online questionnaire (N = 244) that included tests for assessing personality traits (Temperament and Character Inventory; Locus of Control of Behaviour) and moral judgment (Moral Foundations Questionnaire), alongside behavioural economics tasks addressing different facets of risk attitude (loss aversion, risk aversion and delay discounting). We then assessed the extent to which individual variations in these dimensions modulated participants’ compliance with the lockdown norms. When assessing their joint contribution via multiple regressions, lockdown adherence was mostly predicted by internal locus of control, psycho-economic dimensions suggestive of long-sighted and loss-averse attitudes, as well as personality traits related to cautionary behaviour, such as harm avoidance, and the authority moral concern. These findings show that a multi-domain assessment of the factors underlying personal intentions, and thus driving compliance with government measures, can help predict individuals’ actions during health emergencies. This evidence points to factors that should be considered when developing interventions and communication strategies to promote precautionary behaviours.

## 1. Introduction

The COVID-19 outbreak compelled the sudden implementation of measures restricting freedom of movement and social interactions. Several countries worldwide put in place ad hoc norms [1], with different degrees of restrictions ranging from the strong recommendation to avoid crowded places to specific social distancing rules and strict lockdown, depending on the local spread of the contagion and political decisions [2].

Regardless of the severity of their actions, all governments have faced the need of efficiently communicating the risk of infection and its consequences [3, 4]. Italy, for instance, first tackled this issue when the country entered a national lockdown in early March 2020 as the number of new infections and deaths dramatically increased [5]. The challenge continued in subsequent pandemic waves, during which restrictions had to be introduced according to the risk of virus transmission in different regions of the country. This extraordinary situation highlighted the pitfalls of risk communication as an effective tool for raising awareness [6] and increasing compliance in the population [7]. Knowing the severity and probability of adverse outcomes is indeed crucial for appropriate behavioural adjustments, particularly in complex choice settings involving others’, besides one’s own, welfare, and entailing prosocial and moral considerations [8]. Several factors might thus influence risk assessment in different contexts [9], such as the illness or death of a loved one, trust in governmental and scientific institutions, personal knowledge and one own’s set of values and beliefs [10, 11]. The uncertainty of the future and the unpredictability of events, linked to the virus mutations and the consequent measures to limit its spread, have led the population to manifest anxiety and stress-related behaviours [12, 13], such as the unprecedented episodes of panic buying [14, 15]. All these factors are likely to increase people’s difficulties in assessing potential risks [16, 17], and thus in implementing appropriate behaviours to counteract them.

Emergencies, such as the current COVID-19 pandemic, prompted behavioural scientists to examine people’s actual choices in the face of potential consequences and to evaluate the effectiveness of strategies aimed at encouraging adaptive behaviour [18]. Behavioural economics offers tools and insights to investigate individuals’ choices, and promote desired behaviours, even in extraordinary contingencies in which the lack of consistent data (e.g., about the actual risk of contagion) represents a challenge for people’s ability to make choices by weighing individual dispositions and external pressures [19–23]. In the acute lockdown stage, the behavioural effects of the well-known human biases in judgment and decision-making [24, 25] might thus be heightened by the need to filter an unprecedented amount of incoming information from multiple, often contradictory, sources [26]. Even more than in other choice settings, in a pandemic their effect might be also modulated by the awareness of the consequences of one’s own actions on others’ welfare [27]. A sense of “social responsibility” inherent in choices aimed to preserve others, besides oneself, is thus expected to amplify individual differences in decision-making under risk through the modulation by ethical and moral considerations [28].

The endogenous variables shaping the impact of perceived risk on decision-making, and thus possibly modulating behavioural responses to emergency situations and disaster preparedness, include individual dimensions defined by the sense of control over events (Locus of control; [29]), moral dispositions (Theory of Moral Foundations [30–34]) and personality traits [35, 36]. In particular, an internal locus of control appears to buffer COVID-related stress [37] and mental burden [38], additionally promoting medication compliance and health-related behaviour [39, 40]. Previous evidence on the relation between COVID-19 disease concern and sensitivity to moral wrongdoing [41] suggests that adherence to containment measures is also driven by moral values [42]. Finally, personality traits have been shown to reflect adaptive decision-making drivers such as obtaining rewards, avoiding punishments, and managing uncertainty. Indeed, previous neuroeconomic studies have often used the Temperament and Character Inventory (TCI) to investigate the relationship between choice behaviour and temperamental dimensions [43, 44]. All these variables can be expected to modulate the cognitive processes underlying the detection, management, and resolution of typical decisional conflicts such as risk vs. certainty [45], positive vs. negative consequences [17], immediate vs. delayed utility [46], and utilitarian vs. prosocial considerations [47, 48].

An emergency scenario having global health consequences is likely to increase the role of endogenous factors in determining people’s propensity to prioritize public interests above personal risk assessments [49, 50]. Unveiling the psychological and cognitive precursors of the adherence to lockdown might thus provide useful indications for implementing interventions and communication strategies by scientific and political institutions [18]. With the aim of extending our previous evidence on the role of personality traits and moral dispositions in modulating lockdown adherence [51], here we collected measures related to locus of control, risk attitude (risk aversion and loss aversion) and intertemporal preference (delay discounting). Based on previous evidence, we hypothesized that higher levels of compliance with containment measures would be related to a cautious [36] and long-sighted attitude [52, 53], alongside an internal locus of control [54] and a moral disposition towards compliance with the rules and social responsibility [55].

## 2. Materials and methods

### 2.1 Participants

The initial sample included 269 participants who joined the survey between March 30^th^ and May 1^st^, 2020, i.e., in a time window ranging from one week after the start until the end of the full national lockdown in Italy. Since twenty-five participants were excluded for incomplete data, the final sample includes 244 participants (189 females; mean age = 33.05 ± 13.51 years, range: 18–82). Students accounted for 35.69% of the sample, resulting in an average education of 16.36 ± 2.65 years. Table 1 in Results section 3.2 reports the socio-demographic characteristics of the sample.

**Table 1:** Summary of the socio-demographic characteristics of the sample.

**Gender**	Females	168 (68.86%)
	Males	76 (31.14%)
**Age**	Mean (standard deviation)	33.04 (13.54)
	Range	18–82
**Education**	Mean (standard deviation)	16.36 (2.65)
	Secondary school	2 (0.81%)
	High school	69 (28.27%)
	Bachelor	54 (22.13%)
	Master	94 (38.52%)
	Postgraduate	25 (10.24%)
**Occupation**	Student	88 (36.06%)
	Unemployed	5 (2.04%)
	Freelancer	24 (9.83%)
	Employee	41 (16.80%)
	Worker	2 (0.81%)
	Retired	14 (5.73%)
	Health care profession	16 (6.55%)
	Researcher	24 (9.83%)
	Seller	2 (0.81%)
	Agriculture	1 (0.40%)
	Other	27 (11.06%)
**Housing situation**	Alone	31 (12.70%)
	Housemate	27 (11.06%)
	Partner	94 (38.52%)
	Children	41 (16.80%)
	Parents/siblings	93 (38.11%)

### 2.2 Study design

Data were collected via an internet-based survey administered using the online platform LimeSurvey (https://www.limesurvey.org/en/) targeting the Italian adult population. Participants were recruited via social networks (Facebook, Twitter, LinkedIn, Instagram) and word of mouth. To avoid self-selection biases, invitations provided only general information without details about the research goal and hypotheses. In particular, the first page of the questionnaire explained that the survey aimed at assessing the relation between various individual factors and the impact of COVID-19 on behaviour and daily habits. Participants were required to be aged 18 or older, and native or proficient Italian speakers. By completing the survey accurately, they were admitted to a lottery for gift-vouchers worth €12 to 17 depending on the outcome of the psycho-economic tasks. All participants gave their informed consent before starting the survey, by clicking on the “Agree” button placed at the bottom of the first page, and the University of Pavia granted ethical approval for this project. Data were collected without geo-location and stored offline for subsequent data analyses.

### 2.3 Baseline predictors of compliance with the lockdown

#### 2.3.1 Socio-demographic data

The first part of the survey aimed to collect socio-demographic data: age, gender, education, (i.e., number of years in school and highest degree), current occupation and housing situation (i.e., living alone, with relatives or flatmates, etc.).

#### 2.3.2 COVID-19: Attitudes and behaviours

Participants first indicated the number of times they left home in the previous week. Then, through a visual slide ranging from 1 on the left to 10 on the right, they rated their perceived risk of contracting COVID-19 by answering the following question: “How exposed do you feel, on a scale of 1 to 10, to the risk of contracting coronavirus disease?”. Moreover, via different sliders with hidden numerical values, they reported the likelihood of leaving their home, in the following week, for the purpose of: a) outdoor physical activity and b) leisure. We measured these two intended behaviours since they were examples of decisions with a good degree of individual freedom of choice, as they were not necessary or externally mandated actions (like for example purchasing essential goods or going to work). Furthermore, citizens’ behaviour in these domains was the object of great public debate both during and after the lockdown, thus representing an ideal target for interventions/risk communication in case of further waves of infections. Since the same question was asked in relation to the two different purposes across the survey, the numerical value associated with the slider position was made not visible in order to prevent subjects from keeping track of previous answers.

#### 2.3.3 Locus of control

We assessed participants’ perception of being in control over the outcome of their life events with the Italian translation [56] of Craig et al.’s scale [29], consisting of two subscales for internal vs. external locus of control. The difference between the two subscales was then used as a synthetic measure of internal (vs. external) locus of control.

#### 2.3.4 Moral cognition

The Italian translation [57] of the Moral Foundation Questionnaire (MFQ; [58]) was administered to assess personal sensitivity to different aspects of moral cognition in terms of concern for: vulnerable individuals’ harm (harm/care), fairness and social justice (fairness/reciprocity), self-sacrifice for the group (ingroup/loyalty), obedience, leadership, and protection (authority/respect), purity and protection from contamination (purity/sanctity).

#### 2.3.5 Personality

We used the Italian translation [59] of the reduced Temperament and Character Inventory (TCI-56; [60]), including different temperament dimensions: harm avoidance, novelty seeking, reward dependence and persistence, reflecting, respectively, behavioural inhibition/punishment, behavioural activation/reward, social reinforcement/sensitivity to social stimuli, and the tendency to maintain behaviour in extinction conditions.

#### 2.3.6 Temporal preferences

**Fig 1:**
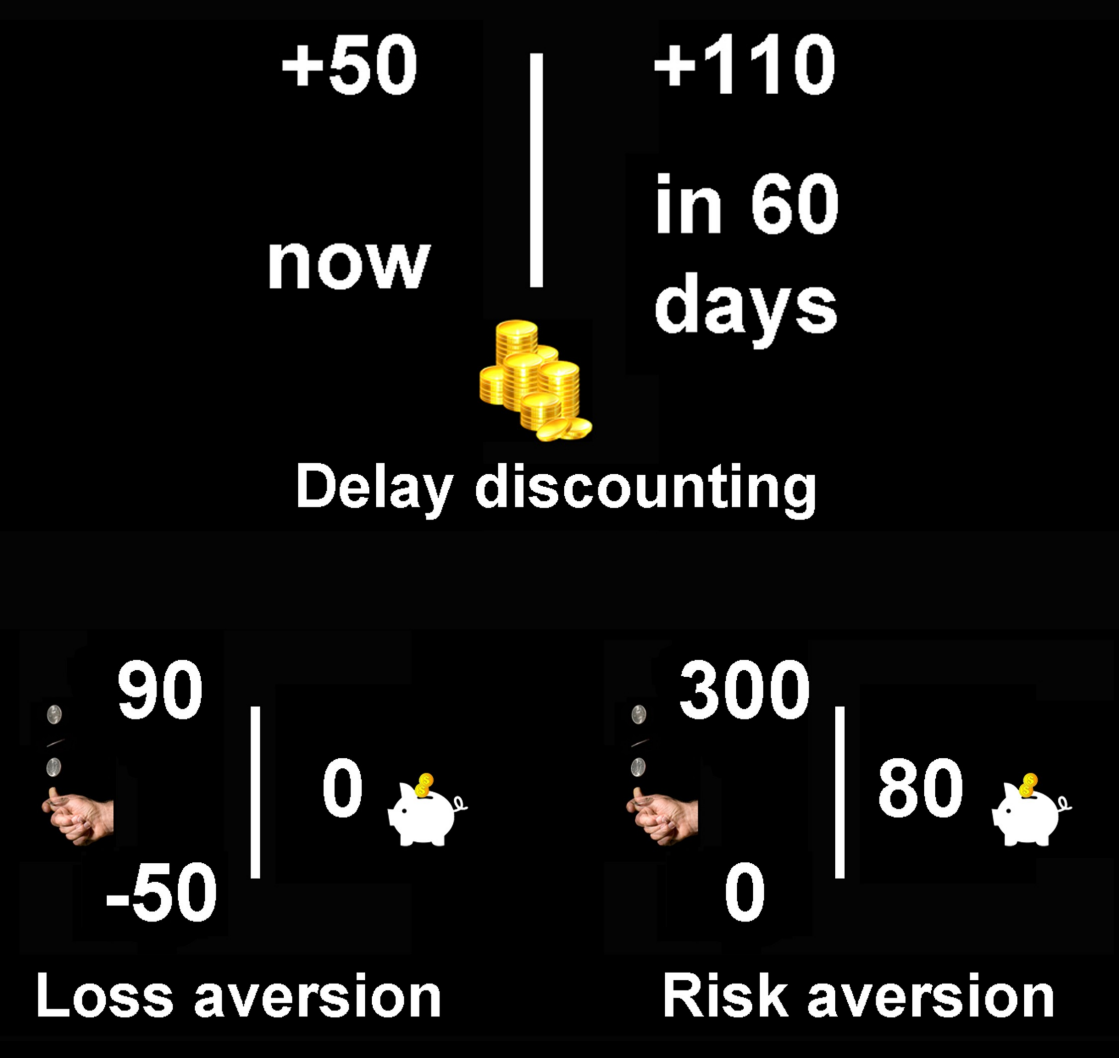
The figure shows example stimuli for the tasks assessing delay discounting (top), loss aversion (bottom-left) and risk aversion (bottom-right).

We used a classical experimental paradigm of intertemporal choice [61] to assess participants’ preferences for options involving costs and benefits occurring at different times. On each trial, they chose between a smaller monetary reward provided immediately and a larger reward paid later. These two options were shown on the left and right halves of the screen (see Fig 1). Participants played 24 trials, entailing different combinations of values of the immediate reward (50, 55, 70, or 90 monetary units), delayed reward (55, 70, 90, or 110 monetary units) and delay duration (7, 30, 60, or 90 days). Choices were made by selecting the left or right option displayed on the screen, corresponding to the immediate or delayed reward. Participants were informed that they would be paid, with a voucher, according to the outcome magnitude and timing of one randomly selected trial.

The degree of discounting of delayed outcomes, reflecting impulsivity vs. self-control [62], was estimated by modelling each subject’s choices with a quasi-hyperbolic discount function, also known as “beta-delta model” [63, 64], as described by McClure et al. [46]. This model explains individual differences in delay-discounting by the combined action of two parameters: “delta” represents the participant’s overall temporal discounting rate (i.e., their tolerance for waiting for rewards), thus indexing a long-sighted preference; “beta” represents the present bias, i.e., the additional amount they discount delayed rewards compared with an immediate one. The beta parameter is thus considered to track the participant’s impulsivity, because it represents the special value placed on the immediate, relative to the delayed, reward (when beta < 1, all future rewards are uniformly down-weighted relative to immediate ones) [46].

#### 2.3.7 Risk aversion

The constraints posed by online surveys, particularly regarding time limitations and lack of supervised training and feedback to participants, do not allow to assess risk attitude with the typical tools of behavioural economics [17, 65–67]. We thus chose to pursue a simpler approach, which has been shown to provide a reliable estimate of risk attitude [68]. Participants took part in a paid lottery with 19 trials. In each trial, they were asked to choose between a variable safe gain or playing a lottery (by flipping a coin) for a fixed larger sum (300 monetary units) (Fig 1). The safe gain increased across trials, in steps of 10, from 0 to 190. Participants were informed that they would be paid, with a voucher, according to the outcome of one randomly selected trial among the accepted ones (i.e., either the safe option or the outcome of the lottery). The individual degree of risk aversion was tracked by the switching point between the safe and risky option along the series of trials. Risk-averse participants were expected to prefer the safe option even when this was below 150 (i.e., the expected value of the lottery), while risk-seekers should prefer the lottery when the safe option is 160 or higher.

#### 2.3.8 Loss aversion

We used a similar approach to assess loss aversion, with 29 trials in which participants chose between the status quo (i.e., the certainty of 0) and a gamble offering equal (fixed at 50%) chances of gaining a variable number of monetary units or losing 50. The possible gain increased across trials, in steps of 5, from 55 to 195. Again, individual differences in loss aversion were indexed by the switching point, between the safe and risky option, along the series of trials. While all gambles should be in principle accepted (because the expected value of the gamble is positive), the overweighting of prospective negative outcomes inherent in loss aversion [17] is expected to reflect in the rejection of gambles up to a gain/loss ratio representing the individual indifference point.

### 2.4 Statistical analyses

We used multiple regression models to assess the contribution of socio-demographic characteristics (age, gender, education), locus of control, sensitivity to MFQ Authority alongside TCI harm avoidance and novelty seeking, and decision-making parameters, to individual variations in different dependent variables concerning COVID-related risk perception and lockdown adherence: perceived risk of contracting COVID-19, number of times participants left home in the previous week, as well as likelihood to leave home in the following week for the purpose of: a) outdoor physical activity and b) leisure. We performed different tests to check the assumptions of the multiple regressions performed for these dependent variables (see Results 3.5, Tables 5 and 6). In all cases, Cook’s distance scores below 1 confirmed the lack of significant outliers which may place undue influence on the model. Variance inflation factors (VIFs) below 2 and tolerance scores above 0.2 allowed excluding multicollinearity among variables. As shown in Tables 5 and 6, indeed, the value of the highest cross-correlation was 0.63, and most of them were below 0.3. A Durbin-Watson score close to 2 confirmed that the values of the residuals of multiple regression models were independent. Instead, only in the first model (with the perceived risk of contracting COVID-19 as dependent variable) the residuals were normally distributed (Kolmogorov-Smirnov test, p>0.2). In the other three models they were not normally distributed (p < .05) due to a right-skewed data distribution. Importantly, it has been shown that linear regressions are valid even for extremely non-normal data (because they do not require any assumption of normal distribution) in sufficiently large samples, which is often the case above 100 participants [69]. Even though a sample of 244 participants is thus expected to result in reliable findings, for these three dependent variables we performed secondary confirmatory analyses using a generalized linear model with the Tweedie distribution, a special case of an exponential right-skewed distribution characterized by data clustering at, or close to, zero, but otherwise continuous [70]. In these analyses we modelled the number of times participants left home in the previous week, as well as the likelihood of leaving home for physical activity or for leisure, as dependent variable, and the significant predictors highlighted by multiple regressions as independent variables.

## 3. Results

### 3.1 Internal consistency

Adequate reliability levels were found for all the subscales of TCI-56, MFQ and locus of control. The internal and external subscales of locus of control were associated with a good internal consistency, as indexed by Cronbach’s alpha values equal to 0.72 and 0.75. We observed a wider range of alpha values, ranging from acceptable (0.63) to very good (0.83) reliability levels, for the five domains of the Moral Foundation Questionnaire (Harm/Care: 0.69; Fairness/Reciprocity: 0.63; Ingroup/Loyalty: 0.77; Authority/Respect: 0.74; Purity/Sanctity: 0.83). These results are fully consistent with the alpha values originally reported by Graham et al. [71], ranging between 0.65 and 0.84, and with the priority to develop items reflecting the broad scope of each moral domain, although at the expense of internal consistency [72]. Finally, we found consistently good alpha values for all the TCI-56 subscales: Harm avoidance (0.75), Novelty seeking (0.73), Reward dependence (0.77), Persistence (0.76), Self-directedness (0.75), Cooperativeness (0.76) and Self-transcendence (0.78). These values are in line with those reported by the authors of the reduced TCI-56 [60].

### 3.2 Socio-demographic and personality variables

The sample socio-demographic and psychological data are reported in Tables 1 and 2, respectively.

**Table 2:** Descriptive statistic for psychological and personality variables.

Psychological and personality variables		Mean (standard deviation)
**Locus of control (LCB)**	Internal—External	-6.20 (5.71)
**Moral Foundation Questionnaire (MFQ)**	Harm	3.86 (0.63)
	Fairness	3.95 (0.49)
	Ingroup	3.01 (0.81)
	Authority	2.61 (0.79)
	Purity	2.05 (0.97)
**Temperament and Character Inventory (TCI)**	Harm Avoidance	24.61 (5.34)
	Novelty Seeking	20.27 (4.51)
	Reward Dependence	28.94 (5.94)
	Persistence	28.47 (4.97)
	Self-directedness	28.75 (5.18)
	Cooperativeness	31.14 (4.66)
	Self-transcendence	20.00 (7.39)

### 3.3 COVID-19-related attitudes and behaviours

Descriptive statistics concerning participants’ actual behaviour and behavioural intentions are reported in Table 3.

**Table 3:** Descriptive statistics for the reported behaviour and behavioural intentions concerning the lockdown.

Behaviour and behavioural intentions		Mean (standard deviation)
Left home in the previous week (number of times)		2.05 (3.86)
Perceived risk (1–10)		4.48 (2.11)
Likelihood to leave home in the next week for (0–100):	physical activity	10.29 (22.61)
	leisure	4.06 (14.51)

### 3.4 Decision-making variables

Participants were on average loss- and risk-averse (see Table 4). In the loss aversion task, the average switching point between accepting and rejecting the gamble corresponded to a gain-loss ratio of 1.99 (i.e., gain = 100 and loss = 50). This value fits with a considerable literature indicating an indifferent ratio close to 2 [17, 73]. In the case of risk aversion, the average switching point between the gamble and the safe option corresponded to a potential gain of 90 (gamble-safe ratio = 4.32). This means that participants started preferring the safe option when its value was well below the gamble expected value (150, corresponding to a gamble-safe ratio = 2 with p = 50%).

**Table 4:** Descriptive statistics for the decision-making variables.

Decision-making variable	Median (standard error)
Loss aversion (switching ratio)	1.99 (0.056)
Risk aversion (switching ratio)	4.32 (0.188)
Delay discounting beta (short-sighted attitude)	0.89 (0.157)
Delay discounting delta (long-sighted attitude)	0.99 (0.156)

As to delay discounting, we confirmed that the dual (beta-delta) parameter model (R^2^ = 0.84) fits the data better than the single-parameter hyperbolic (R^2^ = 0.78) and exponential (R^2^ = 0.74) models. We found an average beta parameter < 1 (beta = 0.89), showing that the sample was biased towards sooner rewards [48].

### 3.5 Variables predicting adherence to lockdown

Multiple regression analyses revealed statistically significant models for all the considered dependent variables (Tables 5 and 6): perceived risk of contracting COVID-19 [R^2^ = 0.124, F(3,240) = 11.276, p<0.00001], number of times leaving home in the previous week [R^2^ = 0.143, F(3,240) = 13.330, p<0.00001], likelihood to leave home for a) physical activity [R^2^ = 0.120, F(6,237) = 5.401, p<0.0001], and b) leisure [R^2^ = 0.101, F(4,239) = 6.690, p<0.0001]. The perceived risk of contracting COVID-19 was predicted by gender (higher in females) and TCI Harm avoidance scores. The number of times participants left home in the previous week was negatively related to an internal locus of control and MFQ Authority, and positively related to age. The likelihood of leaving home for physical activity was positively related to the short-term oriented delay-discounting beta parameter, and negatively related to an internal locus of control, TCI Harm avoidance, MFQ Authority, as well as degree of loss aversion and of the long-term oriented delay-discounting delta parameter. Finally, the likelihood of leaving home for leisure was positively related to the short-term oriented delay-discounting beta parameter, and negatively related to an internal locus of control, TCI Harm avoidance and the long-term oriented delay-discounting delta parameter.

**Table 5:** Multiple regression analysis.

**Multiple Regression **		**Model**			**Independence of residuals**	**Normality of residuals**	**Outliers**		
		**R-square**	**F model**	**p-value**	**Durbin-Watson**	**K-S**	**Cook’s distance **		
		0.124	11.276	<0.00001	1.930	>0.20	all subjects <1		
		**Standardized coefficient**				**Effect size**		**Collinearity**	
**Dependent variable**	**Predictors**	**Beta**	**t**	**p-value**		**partial eta squared**	**observed power**	**VIF**	**tolerance**
Perceived risk of infection	Gender	0.141	2.223	0.027		0.0202	0.600	1.1078	0.903
	TCI Harm Avoidance	0.129	2.026	0.044		0.0168	0.523	1.107	0.903
**Multiple Regression**		**Model**			**Independence of residuals**	**Normality of residuals**	**Outliers**		
		**R-square**	**F model**	**p-value**	**Durbin-Watson**	**K-S**	**Cook’s distance**		
		0.143	13.330	<0.00001	1.783	<0.05	all subjects <1		
		**Standardized coefficient**				**Effect size**		**Collinearity**	
**Dependent variable**	**Predictors**	**Beta**	**T**	**p-value**		**partial eta squared**	**observed power**	**VIF**	**tolerance**
Number of times left home in the previous week	Age	0.267	4.421	<0.00001		0.075	0.993	1.025	0.976
	Locus of control—internal vs. external	-0.238	-3.975	<0.00001		0.062	0.977	1.000	0.999
	MFQ Authority	-0.167	-2.756	0.006		0.031	0.784	1.025	0.976

**Table 6:** Multiple regression analysis.

**Multiple Regression**		**Model**			**Independence of residuals**	**Normality of residuals**	**Outliers **		
		**R-square**	**F model**	**p-value**	**Durbin-Watson**	**K-S**	**Cook’s distance **		
		0.120	5.401	<0.0001	2.041	<0.05	all subjects <1		
		**Standardized coefficient**				**Effect size**		**Collinearity**	
**Dependent variable**	**Predictors**	**Beta**	**t**	**p-value**		**partial eta squared**	**observed power**	**VIF**	**tolerance**
Likelihood of leaving home for physical activity	Locus of control—internal vs. external	-0.137	-2.218	0.027		0.020	0.598	1.040	0.961
	MFQ Authority	-0.131	-2.128	0.034		0.019	0.563	1.015	0.985
	TCI Harm avoidance	-0.132	-2.122	0.035		0.019	0.561	1.046	0.957
	Loss aversion	-0.157	-2.547	0.012		0.027	0.718	1.028	0.973
	Delay discounting—beta (short term oriented)	0.141	2.097	0.037		0.018	0.551	1.224	0.817
	Delay discounting—delta (long term oriented)	-0.239	-3.563	<0.001		0.050	0.944	1.212	0.825
**Multiple Regression**		**Model **			**Independence of residuals**	**Normality of residuals**	**Outliers**		
		**R-square**	**F model**	**p**	**Durbin-Watson**	**K-S**	**Cook’s distance**		
		0.101	6.690	<0.0001	2.107	<0.05	all subjects <1		
		**Standardized coefficient**				**Effect size**		**Collinearity**	
**Dependent variable**	**Predictors**	**Beta**	**t**	**p**		**partial eta squared**	**observed power**	**VIF**	**tolerance**
Likelihood of leaving home for leisure	Locus of control—internal vs. external	-0.123	-1.97	0.049		0.016	0.502	1.039	0.962
	TCI Harm Avoidance	-0.141	-2.264	0.024		0.021	0.616	1.035	0.966
	Delay discounting—beta (short term oriented)	0.163	2.433	0.016		0.024	0.679	1.197	0.835
	Delay discounting—delta (long term oriented)	-0.284	-4.235	<0.0001		0.070	0.988	1.198	0.835

The table reports the significant predictors of the variance of the perceived risk of infection (top) and number of times in which participants left home in the previous week (bottom) based on multiple regressions. For each dependent variable, the statistical values of both the whole model and the single predictors are reported, alongside the results of tests assessing the assumptions for multiple regressions, i.e. presence of outliers (Cook’s distance), multicollinearity (variance inflation factor (VIF) and tolerance), independence of residuals (Durbin-Watson) and normality of residuals (Kolmogorov-Smirnov).

The table reports the significant predictors of the variance of the likelihood of leaving home for physical activity (top) and leisure (bottom) based on multiple regressions. For each dependent variable, the statistical values of both the whole model and the single predictors are reported, alongside the results of tests assessing the assumptions for multiple regressions, i.e. presence of outliers (Cook’s distance), multicollinearity (variance inflation factor (VIF) and tolerance), independence of residuals (Durbin-Watson) and normality of residuals (Kolmogorov-Smirnov).

These results were mostly confirmed by secondary analyses based on generalized models with the Tweedie distribution, which is well suited for right-skewed data distributions. As shown in Table 7, these analyses highlighted the same significant predictors resulting from multiple regressions, with only one exception: TCI Harm avoidance was not confirmed as a significant predictor of the likelihood of leaving home for physical activity and will not be discussed further.

**Table 7:** Generalized regression analysis.

**Tweedie Generalized regression model**						**95% Confidence Interval**	
**Dependent variable**	**Predictors**	**B**	**Standard Error (B)**	**Wald stat**	**p-value**	**Lower**	**Upper**
Number of times left home in the previous week	Age	0.0342	0.005	39.949	<0.000001	0.024	0.045
	Locus of control—internal vs. external	-0.056	0.015	14.817	<0.001	-0.085	-0.028
	MFQ Authority	-0.318	0.104	9.454	0.002	-0.521	-0.115
**Tweedie Generalized regression model**						**95% Confidence Interval**	
**Dependent variable**	**Predictors**	**B**	**Standard Error (B)**	**Wald stat**	**p-value**	**Lower**	**Upper**
Likelihood of leaving home for physical activity	Locus of control—internal vs. external	-0.042	0.020	4.284	0.038	-0.083	-0.002
	MFQ Authority	-0.301	0.144	4.355	0.037	-0.583	-0.018
	TCI Harm Avoidance	-0.040	0.022	3.311	0.068	-0.083	0.003
	Loss aversion	-0.044	0.015	9.011	0.003	-0.072	-0.015
	Delay discounting—beta (short term oriented)	0.245	0.106	5.395	0.020	0.038	0.452
	Delay discounting—delta (long term oriented)	-0.361	0.108	11.087	<0.001	-0.573	-0.148
**Tweedie Generalized regression model**						**95% Confidence Interval**	
**Dependent variable**	**Predictors**	**B**	**Standard Error (B)**	**Wald stat**	**p-value**	**Lower**	**Upper**
Likelihood of leaving home for leisure	Locus of control—internal vs. external	-0.087	0.026	11.160	<0.001	-0.138	-0.036
	TCI Harm Avoidance	-0.104	0.029	13.267	<0.001	-0.160	-0.048
	Delay discounting—beta (short term oriented)	0.453	0.096	22.210	<0.00001	0.264	0.641
	Delay discounting—delta (long term oriented)	-0.576	0.131	19.472	<0.00001	-0.832	-0.320

The table reports the significant predictors of the variance of number of times in which participants left home in the previous week (top), and likelihood of going out for outdoor physical activity (Middle) and leisure (bottom) based on a generalized model with a Tweedie distribution. For each dependent variable, the statistical values of the single significant predictors are reported, alongside their 90% confidence interval.

## 4. Discussion

The COVID-19 pandemic has highlighted the potential impact of individual actions on public health [74]. The dynamics and consequences of the outbreak have also revealed, however, the shortcomings of communication campaigns appearing to neglect the variety of variables mediating their impact on actual risk attitudes and behaviours. In this sense, a full assessment of the psychological elements determining lockdown adherence could considerably improve the outcome of risk communication. We aimed at investigating how much individual differences in locus of control, risk attitude, moral dispositions, and personality factors influenced participants’ adherence to lockdown restrictions. These variables were chosen based on previous evidence of their interdependence, showing for instance a joint modulation of decision-making by risk-aversion and harm avoidance [75], and that behavioural adaptations to emergency situations reflect cautious [36] and long-sighted attitudes [53], but also other-regarding moral dispositions [51, 55]. Specific combinations of these variables were indeed observed to predict individual differences concerning the perceived risk of infection, actual behaviour and behavioural intentions related to lockdown norms.

Internal locus of control, i.e. the individual perception of being in charge, through voluntary actions, of one’s own destiny and life events [76], was found as the most consistent principal predictor of the past and future tendency to comply with the lockdown rules. An internal locus of control was generally predictive of both participants’ actual behaviour in the preceding week and the intention not to leave their homes in the following one, which fits with previous evidence of its connection with health well-being, coping and reappraisal in stressful situations or illnesses [77–79]. Internal locus of control, especially when in health-related settings, is also linked to higher adherence to treatments [80], and might have thus generally supported people’s compliance with lockdown norms. In keeping with this hypothesis, internal and external locus of control have been associated with decreased general mental distress [37] and increased depressive symptoms [81], respectively, during the COVID-19 pandemic.

Along with the perceived control over one’s own life and health, the personal disposition towards immediate or delayed outcomes played a crucial role in the compliance with COVID-19 restrictions. This finding complements previous evidence showing that the typical devaluation of delayed rewards, i.e., “delay-discounting”, also predicts health-related—and not only economic—behaviour [52]. The strong connection between delay-discounting and impulsivity [62], distorted time-perception and sub-optimal decision-making [82] might thus explain why, in the present study, a short-sighted disposition predicted the intention to leave home for both outdoor physical activity and leisure. The fact that these activities are not strictly necessary is likely to reduce constraints when evaluating whether to pursue them or not, thus magnifying the effect of individual differences in impulsive vs. long-sighted dispositions [62, 83].

Also moral cognition predicted participants’ disposition to respect the enforced rules of lockdown. In particular, higher sensitivity to a moral foundation such as authority predicted both actual past behaviour and the intention to break the confinement for outdoor sports. Despite the fact that it does not appear to predict spontaneous prosocial behaviour [84], the legitimacy of authority and the avoidance of sanctions represent an intrinsic forerunners of rule compliance, especially in the face of weak reasons to act differently, like individual outdoor physical activity [51].

The intention of leaving home for physical activity was also significantly predicted by so-called loss aversion, i.e., the overweighting of the negative, over the positive, consequences of choices [17]. The parameters encoded in loss aversion track different facets of a typical cautionary approach which is inclined to prefer the avoidance of negative consequences over the acquisition of equivalent positive ones. This basic principle reflects the association of loss aversion with brain structures driving the avoidance of potential threats via the engagement of aversive affective reactions, such as the amygdala and insula [65, 66]. Most importantly, it provides a framework explaining why people are generally more motivated by penalty frames [85], which are indeed more often applied, compared with reward frames, in behaviour change strategies [86]. The present evidence suggests the effectiveness of loss aversion in modulating behavioural dispositions in complex, real-world, decisional conflicts between oppositely directed motivations concerning personal vs. collective health.

It is worth noting that, the perceived risk of contracting the disease was higher in women–consistent with previous data interpreted as linked to females’ heightened sensitivity [87–89]–and related to the harm avoidance personality trait. This temperament trait is characterized by excessive apprehension, caution, and pessimism [90], and is typically associated with stronger unconscious emotional reactions [91], pain perception [92] and subsequent anticipatory avoidance behaviour, especially of settings that may lead to infection [93, 94]. This trait is also tightly related to the Big Five Inventory neuroticism factor [75, 90, 95, 96], and high levels of neuroticism have been shown to reflect in higher compliance with COVID-19 restrictions [97]. In line with this evidence, the present findings showed that the heightened cautiousness and worrying embodied in the harm avoidance personality trait predicted a decreased likelihood of going out for a relatively unnecessary motive such as leisure.

Overall, these results suggest that distinct facets of the evaluations underlying decision-making predict behavioural dispositions towards different reasons for violating the lockdown. Moreover, they show that the notions and methods of behavioural economics provide valuable insights concerning individuals’ behaviour during the COVID-19 pandemic, and particularly the variables predicting their adherence to social distancing, or rather the willingness to violate the lockdown, for several real-life motives. The present findings might thus inform public health policies and interventions [98], as well as contribute to the development of risk communication plans tailored to different target groups in order to promote appropriate behaviours [18]. For instance, to reduce the severe consequences of infectious outbreaks and related containment efforts, authorities may pursue ad-hoc prevention strategies against impulsive behaviour, problematic personality characteristics and irresponsibility.

The findings of this online study should be considered in light of some limitations. First, the online survey approach required simplified procedures for assessing participants’ risk attitude compared to laboratory settings. Furthermore, we were unable to exclude the potential confounding effect of clinical dimensions such as—for instance—prior history of psychological distress or psychopathologies. While on one hand this was a forced choice during the lockdown, on the other it also allowed a more widespread recruitment and access to a more varied sample. Second, a potential decrease in data quality—possibly resulting in small effect sizes—represents a typical drawback of online procedures compared to controlled experimental settings; however, we addressed this possible issue by undertaking a formal assessment of the consistency/reliability of the data generated from psychometric scales. As to the effect size, further studies comparing online and laboratory studies on the same variables will be required to assess whether these types of effects are inherently small or depend on other factors that are magnified by online data collection, such as individual variability. Finally, inhomogeneous samples are common in online surveys, and therefore cannot be considered representative of the entire population, since they favour young adults over older people. However, while the elderly are at the greatest danger of serious health consequences, young adults provide a highly informative sample for our research topics. In fact, because of their greater work and social commitments, in turn associated with greater risks of mediating untraced virus spread [99], this group may represent an ideal target for propagating and incentivizing health habits, such as mask use, hygienic measures and social distancing, right because of their central social role [18] and proximity to parents and older relatives.

## 5. Conclusions

We highlighted a multifaceted set of psychological variables predicting the perceived risk of contracting COVID-19, and the disposition to respect the lockdown, around the peak of the first outbreak in Italy. Internal locus of control, temporal preference, as well as different facets of decision-making, moral foundations, and personality traits, were shown to play a role in the adherence to the lockdown norms. By unveiling endogenous variables predicting the perceived risk of infection and the likelihood of violating restrictive norms, our findings provide relevant insights that might prove useful in the future management of the pandemic and further public health situations. In a wider perspective, the present results support the relevance of psychology and behavioural economics measures to be used by governmental and scientific entities to shape preventive plans and emergency interventions.

## References

[1] Chaudhry R, Dranitsaris G, Mubashir T, Bartoszko J, Riazi S. A country level analysis measuring the impact of government actions, country preparedness and socioeconomic factors on COVID-19 mortality and related health outcomes. EClinicalMedicine. 2020; 100464. doi: 10.1016/j.eclinm.2020.100464 32838237 PMC7372278

[2] Kenis P, Schol LGC, Kraaij‐Dirkzwager MM, Timen A. Appropriate Governance Responses to Infectious Disease Threats: Developing Working Hypotheses. Risk Hazards Crisis Public Policy. 2019;10: 275–293. doi: 10.1002/rhc3.12176

[3] Betsch C, Wieler LH, Habersaat K, COSMO group. Monitoring behavioural insights related to COVID-19. Lancet Lond Engl. 2020;395: 1255–1256. doi: 10.1016/S0140-6736(20)30729-7 32247323 PMC7163179

[4] Zhang L, Li H, Chen K. Effective Risk Communication for Public Health Emergency: Reflection on the COVID-19 (2019-nCoV) Outbreak in Wuhan, China. Healthcare. 2020;8: 64. doi: 10.3390/healthcare8010064 32245157 PMC7151105

[5] European Centre for Disease Prevention and Control. COVID-19 situation update worldwide, as of 29 July 2020. In: European Centre for Disease Prevention and Control [Internet]. 2020 [cited 29 Jul 2020]. Available: https://www.ecdc.europa.eu/en/geographical-distribution-2019-ncov-cases.

[6] Funk S, Gilad E, Watkins C, Jansen VAA. The spread of awareness and its impact on epidemic outbreaks. Proc Natl Acad Sci. 2009;106: 6872–6877. doi: 10.1073/pnas.0810762106 19332788 PMC2672559

[7] Covello VT. Best Practices in Public Health Risk and Crisis Communication. J Health Commun. 2003;8: 5–8. doi: 10.1080/713851971 14692565

[8] Smillie LD, Lawn ECR, Zhao K, Perry R, Laham SM. Prosociality and morality through the lens of personality psychology. Aust J Psychol. 2019;71: 50–58. doi: 10.1111/ajpy.12229

[9] Lanciano T, Graziano G, Curci A, Costadura S, Monaco A. Risk Perceptions and Psychological Effects During the Italian COVID-19 Emergency. Front Psychol. 2020;11: 580053. doi: 10.3389/fpsyg.2020.580053 33071920 PMC7533588

[10] Dryhurst S, Schneider CR, Kerr J, Freeman ALJ, Recchia G, van der Bles AM, et al. Risk perceptions of COVID-19 around the world. J Risk Res. 2020;23: 994–1006. doi: 10.1080/13669877.2020.1758193

[11] Brown R, Coventry L, Pepper G. COVID-19: the relationship between perceptions of risk and behaviours during lockdown. J Public Health. 2021 [cited 22 Oct 2021]. doi: 10.1007/s10389-021-01543-9 34007783 PMC8118375

[12] Flesia L, Monaro M, Mazza C, Fietta V, Colicino E, Segatto B, et al. Predicting Perceived Stress Related to the Covid-19 Outbreak through Stable Psychological Traits and Machine Learning Models. PsyArXiv; 2020 Apr. doi: 10.31234/osf.io/yb2h8 PMC7603217 33086558

[13] Amanzio M, Canessa N, Bartoli M, Cipriani GE, Palermo S, Cappa SF. Lockdown Effects on Healthy Cognitive Aging During the COVID-19 Pandemic: A Longitudinal Study. Front Psychol. 2021;12: 685180. doi: 10.3389/fpsyg.2021.685180 34108923 PMC8180921

[14] Chua G, Yuen KF, Wang X, Wong YD. The Determinants of Panic Buying during COVID-19. Int J Environ Res Public Health. 2021;18: 3247. doi: 10.3390/ijerph18063247 33801077 PMC8003931

[15] Islam T, Pitafi AH, Arya V, Wang Y, Akhtar N, Mubarik S, et al. Panic buying in the COVID-19 pandemic: A multi-country examination. J Retail Consum Serv. 2021;59: 102357. doi: 10.1016/j.jretconser.2020.102357

[16] Campos-Mercade P, Meier A, Schneider F, Wengström E. Prosociality Predicts Health Behaviors during the COVID-19 Pandemic. Rochester, NY: Social Science Research Network; 2020 May. Report No.: ID 3604094. doi: 10.2139/ssrn.3604094 PMC7842154 33531719

[17] Kahneman D, Tversky A. Prospect Theory: An Analysis of Decision under Risk. Econometrica. 1979;47: 263–91.

[18] Bavel JJV, Baicker K, Boggio PS, Capraro V, Cichocka A, Cikara M, et al. Using social and behavioural science to support COVID-19 pandemic response. Nat Hum Behav. 2020;4: 460–471. doi: 10.1038/s41562-020-0884-z 32355299

[19] Linnemayr S, O’Hanlon C, Uscher-Pines L, Van Abel K, Nelson C. Using Insights From Behavioral Economics to Strengthen Disaster Preparedness and Response. Disaster Med Public Health Prep. 2016;10: 768–774. doi: 10.1017/dmp.2016.29 27188246

[20] Linnemayr S, Rice T. Insights From Behavioral Economics to Design More Effective Incentives for Improving Chronic Health Behaviors, With an Application to Adherence to Antiretrovirals. J Acquir Immune Defic Syndr 1999. 2016;72: e50–52. doi: 10.1097/QAI.0000000000000972 26918543 PMC4866888

[21] Liu W, Li L. Emergency decision-making combining cumulative prospect theory and group decision-making. Granul Comput. 2019;4: 39–52. doi: 10.1007/s41066-018-0086-5

[22] Zhang Z-X, Wang L, Wang Y-M. An Emergency Decision Making Method Based on Prospect Theory for Different Emergency Situations. Int J Disaster Risk Sci. 2018;9: 407–420. doi: 10.1007/s13753-018-0173-x

[23] Bartoli M, Canessa N, Cipriani GE, Cappa SF, Amanzio M. The Role of Neuropsychological Factors in Perceived Threat of SARS-CoV-2 in Healthy Ageing. Int J Environ Res Public Health. 2021;18: 5847. doi: 10.3390/ijerph18115847 34072431 PMC8198099

[24] Kahneman D, Slovic P, Tversky A. Judgment Under Uncertainty: Heuristics and Biases. Cambridge University Press; 1982. 10.1126/science.185.4157.1124 17835457

[25] Tversky A, Kahneman D. Judgment under Uncertainty: Heuristics and Biases. Science. 1974;185: 1124–1131. doi: 10.1126/science.185.4157.1124 17835457

[26] Hilbert M. Toward a synthesis of cognitive biases: How noisy information processing can bias human decision making. Psychol Bull. 2012;138: 211–237. doi: 10.1037/a0025940 22122235

[27] Bolton GE, Ockenfels A, Stauf J. Social responsibility promotes conservative risk behavior. Eur Econ Rev. 2015;74: 109–127. doi: 10.1016/j.euroecorev.2014.10.002

[28] van Baar JM, Chang LJ, Sanfey AG. The computational and neural substrates of moral strategies in social decision-making. Nat Commun. 2019;10: 1483. doi: 10.1038/s41467-019-09161-6 30940815 PMC6445121

[29] Craig AR, Franklin JA, Andrews G. A scale to measure locus of control of behaviour. Br J Med Psychol. 1984;57: 173–180. doi: 10.1111/j.2044-8341.1984.tb01597.x 6743598

[30] Morality Haidt J. Perspect Psychol Sci. 2008 [cited 29 Jul 2020]. Available: https://journals.sagepub.com/doi/10.1111/j.1745-6916.2008.00063.x.

[31] Slovic P, Peters E, Finucane ML, Macgregor DG. Affect, risk, and decision making. Health Psychol Off J Div Health Psychol Am Psychol Assoc. 2005;24: S35–40. doi: 10.1037/0278-6133.24.4.S35 16045417

[32] Mishra S, Suar D, Paton D. Is Externality a Mediator of Experience–Behaviour and Information–Action Hypothesis in Disaster Preparedness? J Pac Rim Psychol. 2009;3: 11–19. doi: 10.1375/prp.3.1.11

[33] Mishra S, Mazumdar S. Psychology of Disaster Preparedness. Ecopsychology. 2015;7: 211–223. doi: 10.1089/eco.2015.0006

[34] Qian K, Yahara T. Mentality and behavior in COVID-19 emergency status in Japan: Influence of personality, morality and ideology. Hashimoto K, editor. PLOS ONE. 2020;15: e0235883. doi: 10.1371/journal.pone.0235883 32649687 PMC7351180

[35] Cloninger CR, editor. The temperament and character inventory (TCI): a guide to its development and use. 1st ed. St. Louis, Mo: Center for Psychobiology of Personality, Washington University; 1994.

[36] Iennaco D, Messina M, Moretto E, DellrOrco S, Costa V, Sperandeo R, et al. Decision-making Styles and Personality Traits: A pilot study on the predictive capacity of the TCI regarding the quality of the decision. 2018 9th IEEE International Conference on Cognitive Infocommunications (CogInfoCom). Budapest, Hungary: IEEE; 2018. pp. 000305–000312. doi: 10.1109/CogInfoCom.2018.8639866

[37] Krampe H, Danbolt LJ, Haver A, Stålsett G, Schnell T. Locus of control moderates the association of COVID-19 stress and general mental distress: Results of a cross-sectional survey in two large samples from Norway, Germany, and Austria. In Review; 2021 May. doi: 10.21203/rs.3.rs-479681/v1 PMC8419811 34488667

[38] Misamer M, Signerski-Krieger J, Bartels C, Belz M. Internal Locus of Control and Sense of Coherence Decrease During the COVID-19 Pandemic: A Survey of Students and Professionals in Social Work. Front Sociol. 2021;6: 705809. doi: 10.3389/fsoc.2021.705809 34604376 PMC8479157

[39] Náfrádi L, Nakamoto K, Schulz PJ. Is patient empowerment the key to promote adherence? A systematic review of the relationship between self-efficacy, health locus of control and medication adherence. Asnani MR, editor. PLOS ONE. 2017;12: e0186458. doi: 10.1371/journal.pone.0186458 29040335 PMC5645121

[40] Kothari R. Correlates of Compliance With COVID-19 Prevention Guidelines: Risk Propensity, Locus of Control, Intolerance of Uncertainty. Int J Indian Psychol. 2021;9. doi: 10.25215/0902.057

[41] Henderson RK, Schnall S. Disease and Disapproval: COVID-19 Concern is Related to Greater Moral Condemnation. Evol Psychol. 2021;19: 147470492110215. doi: 10.1177/14747049211021524 34112018 PMC10358411

[42] Díaz R, Cova F. Reactance, morality, and disgust: the relationship between affective dispositions and compliance with official health recommendations during the COVID-19 pandemic. Cogn Emot. 2021; 1–17. doi: 10.1080/02699931.2021.1941783 34132171

[43] Nagatsuka M. The Effect of Temperament and Character on Bidding Behavior. SSRN Electron J. 2015 [cited 28 Sep 2020]. doi: 10.2139/ssrn.2677487

[44] Yang B, Lester D, Spinella M. Neurotransmitter-Related Personality Traits and Money Attitudes: A Study in Neuroeconomics. Psychol Rep. 2006;98: 21–22. doi: 10.2466/pr0.98.1.21-22 16673945

[45] Oliver A. Your money and your life: Risk attitudes over gains and losses. J Risk Uncertain. 2018;57: 29–50. doi: 10.1007/s11166-018-9284-4

[46] McClure SM, Laibson D, Loewenstein G, Cohen J. Separate Neural Systems Value Immediate and Delayed Monetary Rewards. Science. 2004;306: 503–7. doi: 10.1126/science.1100907 15486304

[47] Greene JD. An fMRI Investigation of Emotional Engagement in Moral Judgment. Science. 2001;293: 2105–2108. doi: 10.1126/science.1062872 11557895

[48] Green L, Myerson J. A Discounting Framework for Choice With Delayed and Probabilistic Rewards. Psychol Bull. 2004;130: 769–792. doi: 10.1037/0033-2909.130.5.769 15367080 PMC1382186

[49] Shi R, Qi W, Ding Y, Liu C, Shen W. Under what circumstances is helping an impulse? Emergency and prosocial traits affect intuitive prosocial behavior. Personal Individ Differ. 2020;159. doi: 10.1016/j.paid.2020.109828

[50] Zaki J. Catastrophe Compassion: Understanding and Extending Prosociality Under Crisis. Trends Cogn Sci. 2020;24: 587–589. doi: 10.1016/j.tics.2020.05.006 32410822 PMC7221394

[51] Lo Presti S, Mattavelli G, Canessa N, Gianelli C. Psychological precursors of individual differences in COVID-19 lockdown adherence: Moderated-moderation by personality and moral cognition measures. Personal Individ Differ. 2021;182: 111090. doi: 10.1016/j.paid.2021.111090 PMC9756798 36540872

[52] Daugherty J, Brase G. Taking time to be healthy: Predicting health behaviors with delay discounting and time perspective. Personal Individ Differ. 2010;48: 202–207. doi: 10.1016/j.paid.2009.10.007

[53] Lawless L, Drichoutis A, Nayga R. Time preferences and health behaviour: a review. Agric Food Econ. 2013;1: 1–19.

[54] Chebat J-C. Social Responsibility, Locus of Control, and Social Class. J Soc Psychol. 1986;126: 559–561. doi: 10.1080/00224545.1986.9713626

[55] O’Grady T, Vandegrift D, Wolek M, Burr G. On the determinants of other-regarding behavior: Field tests of the moral foundations questionnaire☆,☆☆,★,★★. J Res Personal. 2019;81: 224–237. doi: 10.1016/j.jrp.2019.06.008

[56] Farma T, Cortinovis I. Un questionario sul “Locus of Control”: suo utilizzo nel contesto italiano. Ric Psicoter. 2001;1.

[57] Bobbio A, Nencini A, Sarrica M. Il Moral Foundation Questionnaire: Analisi della struttura fattoriale della versione italiana. G Psicol. 2011;5: 7–18.

[58] Graham J, Haidt J, Nosek B. The Moral Foundations Questionnaire. 2008. Available: www.moralfoundations.org.

[59] Fossati A, Cloninger CR, Villa D, Borroni S, Grazioli F, Giarolli L, et al. Reliability and validity of the Italian version of the Temperament and Character Inventory-Revised in an outpatient sample. Compr Psychiatry. 2007;48: 380–387. doi: 10.1016/j.comppsych.2007.02.003 17560961

[60] Adan A, Serra-Grabulosa JM, Caci H, Natale V. A reduced Temperament and Character Inventory (TCI-56). Psychometric properties in a non-clinical sample. Personal Individ Differ. 2009;46: 687–692. doi: 10.1016/j.paid.2009.01.023

[61] Frederick S, Loewenstein G, O’Donoghue T. Time Discounting and Time Preference: A Critical Review. J Econ Lit. 2002;40: 351–401. doi: 10.1257/002205102320161311

[62] Moreira D, Barbosa F. Delay Discounting in Impulsive Behavior: A Systematic Review. Eur Psychol. 2019;24: 312–321. doi: 10.1027/1016-9040/a000360

[63] Laibson D. Golden Eggs and Hyperbolic Discounting. Q J Econ. 1997;112: 443–478. doi: 10.1162/003355397555253

[64] Phelps ES, Pollak RA. On Second-Best National Saving and Game-Equilibrium Growth. Rev Econ Stud. 1968;35: 185–199. doi: 10.2307/2296547

[65] Canessa N, Crespi C, Motterlini M, Baud-Bovy G, Chierchia G, Pantaleo G, et al. The Functional and Structural Neural Basis of Individual Differences in Loss Aversion. J Neurosci. 2013;33: 14307–14317. doi: 10.1523/JNEUROSCI.0497-13.2013 24005284 PMC6618376

[66] Canessa N, Crespi C, Baud-Bovy G, Dodich A, Falini A, Antonellis G, et al. Neural markers of loss aversion in resting-state brain activity. NeuroImage. 2017;146: 257–265. doi: 10.1016/j.neuroimage.2016.11.050 27884798

[67] Tom S, Fox C, Trepel C, Poldrack R. The Neural Basis of Loss Aversion in Decision-Making Under Risk. Science. 2007;315: 515–8. doi: 10.1126/science.1134239 17255512

[68] Dohmen T, Falk A, Heckman J, Huffman D, Schupp J, Sunde U, et al. Individual Risk Attitudes: Measurement, Determinants, And Behavioral Consequences. J Eur Econ Assoc. 2011;9: 522–550. doi: 10.1111/j.1542-4774.2011.01015.x

[69] Lumley T, Diehr P, Emerson S, Chen L. The Importance of the Normality Assumption in Large Public Health Data Sets. Annu Rev Public Health. 2002;23: 151–169. doi: 10.1146/annurev.publhealth.23.100901.140546 11910059

[70] Tweedie MCK. An index which distinguishes between some important exponential families. Statistics: Applications and new directions Proceedings of the Indian Statistical Institute Golden Jubilee International Conference. Calcutta: Indian Statistical Institute: J. K. Ghosh & J. Roy (Eds.); 1984. pp. 579–604.

[71] Graham J, Nosek BA, Haidt J, Iyer R, Koleva S, Ditto PH. Mapping the moral domain. J Pers Soc Psychol. 2011;101: 366–385. doi: 10.1037/a0021847 21244182 PMC3116962

[72] Zhang H, Hook JN, Johnson KA. Moral Foundations Questionnaire. In: Zeigler-Hill V, Shackelford TK, editors. Encyclopedia of Personality and Individual Differences. Cham: Springer International Publishing; 2016. pp. 1–3. doi: 10.1007/978-3-319-28099-8_1252–1

[73] Lee S, Lee MJ, Kim BW, Gilman JM, Kuster JK, Blood AJ, et al. The Commonality of Loss Aversion across Procedures and Stimuli. Marshall JAR, editor. PLOS ONE. 2015;10: e0135216. doi: 10.1371/journal.pone.0135216 26394306 PMC4579072

[74] Brooks SK, Webster RK, Smith LE, Woodland L, Wessely S, Greenberg N, et al. The psychological impact of quarantine and how to reduce it: rapid review of the evidence. The Lancet. 2020;395: 912–920. doi: 10.1016/S0140-6736(20)30460-8 32112714 PMC7158942

[75] Paulus MP, Rogalsky C, Simmons A, Feinstein JS, Stein MB. Increased activation in the right insula during risk-taking decision making is related to harm avoidance and neuroticism. NeuroImage. 2003;19: 1439–1448. doi: 10.1016/s1053-8119(03)00251-9 12948701

[76] Rotter JB. Social learning and clinical psychology. Englewood Cliffs: Prentice-Hall, Inc; 1954. doi: 10.1037/10788-000

[77] Hickie I, Davenport T, Wakefield D, Vollmer-Conna U, Cameron B, Vernon SD, et al. Post-infective and chronic fatigue syndromes precipitated by viral and non-viral pathogens: prospective cohort study. BMJ. 2006;333: 575. doi: 10.1136/bmj.38933.585764.AE 16950834 PMC1569956

[78] Parkes KR. Locus of control, cognitive appraisal, and coping in stressful episodes. J Pers Soc Psychol. 1984;46. Available: https://ora.ox.ac.uk/objects/uuid:af71e25e-cbed-4cc8-9f9a-ac8032bca697. 10.1037//0022-3514.46.3.655 6707867

[79] Roddenberry A, Renk K. Locus of Control and Self-Efficacy: Potential Mediators of Stress, Illness, and Utilization of Health Services in College Students. Child Psychiatry Hum Dev. 2010;41: 353–70. doi: 10.1007/s10578-010-0173-6 20204497

[80] Anna Basinska M, Andruszkiewicz A. Health Locus of Control in Patients With Graves-Basedow Disease and Hashimoto Disease and Their Acceptance of Illness. Int J Endocrinol Metab. 2012;10: 537–542. doi: 10.5812/ijem.3932 23843816 PMC3693625

[81] Sigurvinsdottir R, Thorisdottir IE, Gylfason HF. The Impact of COVID-19 on Mental Health: The Role of Locus on Control and Internet Use. Int J Environ Res Public Health. 2020;17: 6985. doi: 10.3390/ijerph17196985 32987750 PMC7579380

[82] Franken IHA, van Strien JW, Nijs I, Muris P. Impulsivity is associated with behavioral decision-making deficits. Psychiatry Res. 2008;158: 155–163. doi: 10.1016/j.psychres.2007.06.002 18215765

[83] Stam CH, van der Veen FM, Franken IHA. Individual differences in time estimation are associated with delay discounting and alcohol use. Curr Psychol. 2020 [cited 2 Aug 2021]. doi: 10.1007/s12144-020-00899-7

[84] O’Grady T, Vandegrift D. Moral foundations and decisions to donate bonus to charity: Data from paid online participants in the United States. Data Brief. 2019;25: 104331. doi: 10.1016/j.dib.2019.104331 31463347 PMC6706768

[85] Gächter S, Orzen H, Renner E, Starmer C. Are Experimental Economists Prone to Framing Effects? A Natural Field Experiment. J Econ Behav Organ. 2009;70. doi: 10.1016/j.jebo.2007.11.003

[86] Hameleers M. Prospect Theory in Times of a Pandemic: The Effects of Gain versus Loss Framing on Policy Preferences and Emotional Responses During the 2020 Coronavirus Outbreak. SocArXiv; 2020 Apr. doi: 10.31235/osf.io/7pykj

[87] Brug J, Aro AR, Oenema A, de Zwart O, Richardus JH, Bishop GD. SARS Risk Perception, Knowledge, Precautions, and Information Sources, the Netherlands. Emerg Infect Dis. 2004;10: 1486–1489. doi: 10.3201/eid1008.040283 15496256 PMC3320399

[88] Ding Y, Du X, Li Q, Zhang M, Zhang Q, Tan X, et al. Risk perception of coronavirus disease 2019 (COVID-19) and its related factors among college students in China during quarantine. Yi S, editor. PLOS ONE. 2020;15: e0237626. doi: 10.1371/journal.pone.0237626 32790791 PMC7425914

[89] Yang S, Cho S-I. Middle East respiratory syndrome risk perception among students at a university in South Korea, 2015. Am J Infect Control. 2017; S0196655317301347. doi: 10.1016/j.ajic.2017.02.013 28385465 PMC7115287

[90] De Fruyt F, Van De Wiele L, Van Heeringen C. Cloninger’s Psychobiological Model of Temperament and Character and the Five-Factor Model of Personality. Personal Individ Differ. 2000;29: 441–452. doi: 10.1016/S0191-8869(99)00204-4

[91] Yoshino A, Kimura Y, Yoshida T, Takahashi Y, Nomura S. Relationships between temperament dimensions in personality and unconscious emotional responses. Biol Psychiatry. 2005;57: 1–6. doi: 10.1016/j.biopsych.2004.09.027 15607293

[92] Pud D, Eisenberg E, Sprecher E, Rogowski Z, Yarnitsky D. The tridimensional personality theory and pain: harm avoidance and reward dependence traits correlate with pain perception in healthy volunteers. Eur J Pain. 2004;8: 31–38. doi: 10.1016/S1090-3801(03)00065-X 14690672

[93] Duncan LA, Schaller M, Park JH. Perceived vulnerability to disease: Development and validation of a 15-item self-report instrument. Personal Individ Differ. 2009;47: 541–546. doi: 10.1016/j.paid.2009.05.001

[94] Oosterhoff B, Shook NJ, Iyer R. Disease avoidance and personality: A meta-analysis. J Res Personal. 2018;77: 47–56. doi: 10.1016/j.jrp.2018.09.008

[95] McCrae RR, Costa PT. Validation of the five-factor model of personality across instruments and observers. J Pers Soc Psychol. 1987;52: 81–90. doi: 10.1037//0022-3514.52.1.81 3820081

[96] McCrae RR, John OP. An Introduction to the Five-Factor Model and Its Applications. J Pers. 1992;60: 175–215. doi: 10.1111/j.1467-6494.1992.tb00970.x 1635039

[97] Kohút M, Kohútová V, Halama P. Big Five predictors of pandemic-related behavior and emotions in the first and second COVID-19 pandemic wave in Slovakia. Personal Individ Differ. 2021;180: 110934. doi: 10.1016/j.paid.2021.110934 34629578 PMC8487295

[98] Soofi M, Najafi F, Karami-Matin B. Using Insights from Behavioral Economics to Mitigate the Spread of COVID-19. Appl Health Econ Health Policy. 2020;18: 345–350. doi: 10.1007/s40258-020-00595-4 32435987 PMC7239690

[99] Dowd JB, Andriano L, Brazel DM, Rotondi V, Block P, Ding X, et al. Demographic science aids in understanding the spread and fatality rates of COVID-19. Proc Natl Acad Sci. 2020;117: 9696–9698. doi: 10.1073/pnas.2004911117 32300018 PMC7211934

